# Maternal hypertensive disorders during pregnancy and their link to childhood asthma: a systematic review and meta-analysis

**DOI:** 10.3389/fped.2025.1659105

**Published:** 2025-12-08

**Authors:** Yanyun Jia, Yajun He, Chuchu Ai, Yan Liu, Kui Zhang

**Affiliations:** 1 Department of Respiratory Medicine, The First Affiliated Hospital of Xi'an Medical University, Xi’ an, China; 2 Department of Gynecology, The Second Clinical Medical College of Lanzhou University, Lanzhou, China; 3 Department of Gynecology, The First Affiliated Hospital of Xi'an Medical University, Xi’ an, China; 4 The First Clinical Medical College of Lanzhou University, Lanzhou, China

**Keywords:** hypertensive disorders of pregnancy (HDP), preeclampsia, gestational hypertension (GH), childhood asthma, meta-analysis

## Abstract

**Objective:**

The association between Hypertensive Disorders of Pregnancy (HDP) and childhood asthma remains controversial, with limited systematic synthesis. This study aimed to integrate global observational evidence to evaluate the relationship between HDP [and its subtypes, pre-eclampsia (PE) and gestational hypertension (GH)] and offspring asthma risk, while exploring sources of heterogeneity.

**Methods:**

Following PRISMA 2020 and MOOSE guidelines, 20 observational studies (*n* = 9,447,593) were identified via systematic searches in Cochrane Library, PubMed, Web of Science, and ScienceDirect (up to January 2025). Random-effects meta-analysis pooled odds ratios (ORs), with subgroup analyses for HDP subtypes, geographic regions, and offspring age. Quality was assessed using the modified Newcastle-Ottawa Scale (mNOS) for cohorts and AHRQ checklist for cross-sectional studies.

**Results:**

HDP exposure significantly increased childhood asthma risk (pooled OR = 1.20, 95% CI = 1.14–1.26, *P* < 0.00001). PE was associated with asthma (OR = 1.20, 95% CI = 1.13–1.27), but GH was not (OR = 1.15, 95% CI = 0.99–1.32). Stratified analyses revealed increased risk in European (OR = 1.20) and North American (OR = 1.62) populations, and in infants (0–2 years, OR = 1.27) and school-aged children (7–12 years, OR = 1.22), but not in Asian populations or 2–6-year-olds. Sensitivity analyses confirmed result robustness.

**Conclusion:**

HDP (particularly PE) exposure is significantly associated with increased offspring asthma risk, with age- and region-specific heterogeneity. Future multicenter cohorts should validate causality and explore subtype-specific mechanisms and interventions for early asthma prevention.

**Systematic Review Registration:**

PROSPERO 2025 CRD420251052620.

## 1 Introduction

Hypertensive disorders of pregnancy (HDP), a group of pregnancy-specific vascular complications with a global prevalence of 5%–15%, are clinically categorized into preeclampsia (PE) and gestational hypertension (GH) (1, 2). These disorders are defined by new-onset hypertension (systolic blood pressure ≥140 mmHg and/or diastolic blood pressure ≥90 mmHg) after 20 weeks of gestation, with or without multi-organ dysfunction (3). Emerging evidence suggests that the long-term impacts of HDP on offspring health extend beyond the perinatal period, with particular attention to its potential pathogenic link to childhood asthma (4–6).

Epidemiological studies indicate that HDP may elevate offspring asthma risk through placental dysfunction, systemic inflammation, and oxidative stress, which disrupt fetal lung morphogenesis and immune programming (7). However, significant heterogeneity exists across studies: prospective cohorts report a 9%–78% increased asthma risk in HDP-exposed offspring (6, 8–17), whereas registry-based analyses fail to demonstrate significant associations (4, 5, 18–22). These discrepancies highlight the critical influence of methodological variations in exposure assessment and confounding control. Mechanistically, HDP-driven biological perturbations may impair respiratory development through multiple pathways (23–25): (1) maternal pro-inflammatory cytokines (TNF-α, IL-6) and anti-angiogenic markers (sFlt-1) traverse the placental interface, suppressing alveolar septation and promoting Th2 immune polarization; (2) epigenetic reprogramming (DNA methylation anomalies, histone modifications) persistently dysregulates transcription of lung development genes (e.g., FOXP3, IL-4); (3) severe PE-associated fetal growth restriction alters pulmonary vascular remodeling and matrix metalloproteinase expression, amplifying susceptibility to postnatal environmental triggers. Despite these advances, systematic reviews remain limited by geographic bias, insufficient quantification of methodological heterogeneity, and outdated evidence synthesis (7, 26).

This study integrates global observational evidence up to 2025 (20 studies, *n* = 9,447,593) to address key questions: (1) Does a dose-response relationship exist between HDP and offspring asthma, and do clinical subtypes (PE vs. GH) exhibit effect heterogeneity? (2) How do exposure ascertainment methods (medical record validation vs. self-report), confounding adjustment strategies (maternal asthma history, tobacco exposure, and study designs (cohort vs. cross-sectional) influence effect estimates?

## 2 Method

### 2.1 Study design and literature search

This study strictly adhered to the Preferred Reporting Items for Systematic Reviews and Meta-Analyses (PRISMA 2020) guidelines and the Meta-analysis of Observational Studies in Epidemiology (MOOSE) checklist. Registered in the International Prospective Register of Systematic Reviews (PROSPERO 2025 CRD420251052620).

#### 2.1.1 Search strategy

A systematic multistage search strategy was implemented by two independent investigators (Yan Liu and Chuchu Ai) through a standardized four-phase process. First, we conducted cross-database searches in Cochrane Library, PubMed, Web of Science, and Elsevier ScienceDirect, covering from database inception to January 2025 with auto-alert functions for emerging studies. Second, structured search terms were developed using Medical Subject Headings (MeSH) combined with free-text keywords. The detailed search terms are as follows: (1) MeSH Terms: Hypertension, Pregnancy-Induced; Childhood Asthma. (2) Free-Text Keywords: Hypertension, Pregnancy Induced, Pregnancy-Induced Hypertension, Gestational Hypertension, Hypertension, Gestational, Pregnancy Induced Hypertension, Hypertensions, Pregnancy Induced, Induced Hypertension, Pregnancy, Induced Hypertensions, Pregnancy, Transient Hypertension, Pregnancy, Hypertension, Pregnancy Transient, Pregnancy Transient Hypertension, Asthmas, Asthma, Bronchial and Bronchial Asthma. (3) Boolean Combination: “MeSH Terms” OR “Free-Text Keywords”. Third, language filters were applied to include only peer-reviewed English clinical studies, excluding non-English publications to mitigate cross-language bias. Finally, two validation mechanisms ensured literature completeness: 1) backward citation tracking of included studies; 2) searches in https://www.ClinicalTrials.gov and conference proceedings for gray literature, with direct author contact for unpublished data.

#### 2.1.2 Eligibility criteria

PICOS framework guided study selection:

Population (P): Clinically diagnosed HDP/PE/GH patients and offspring per ISSHP 2018 criteria (HDP: SBP ≥140 mmHg and/or DBP ≥90 mmHg; PE required new-onset hypertension after 20 weeks with end-organ damage).
Control (C): Normotensive pregnant women (BP <140/90 mmHg) with matched offspring.
Outcomes (O): Primary endpoint: ISACC-defined childhood asthma; Secondary endpoints included wheezing frequency (≥3 episodes/year), lung function (FEV1/FVC <0.8), and treatment intensity (GINA classification).
Study design (S): Prospective/retrospective cohort studies.

Exclusion criteria encompassed non-original research, duplicate datasets (retaining largest samples), and studies with inaccessible key data (after three contact attempts). The selection process was documented through a PRISMA flow diagram.

### 2.2 Data extraction and quality assessment

A double-blind arbitration system was employed for data management. Two researchers independently extracted data via Covidence system, including: Study characteristics; Exposure parameters; Outcome measures; Confounding controls. Discrepancies were resolved by a third reviewer (Yanyun Jia).

Quality assessment utilized evidence-based tools: Cohort studies were evaluated using the modified Newcastle-Ottawa Scale (mNOS, 9-point scale) for selection bias (3 items), comparability (2 items), and outcome assessment (3 items), with ≥7 indicating high quality. Cross-sectional studies were assessed via AHRQ 11-item checklist. All evaluations underwent double-blind independent scoring with inter-rater reliability verified by Kappa statistics (*κ* = 0.85, *P* < 0.001).

### 2.3 Statistical analysis

Evidence synthesis followed the GRADE framework. All included studies adjusted for at least three of the following confounders: maternal age, pre-pregnancy BMI, smoking during pregnancy, maternal education level, parity, gestational age at delivery, offspring sex, maternal asthma history, diabetes mellitus, and socioeconomic status. Two-stage meta-analysis was performed using RevMan 5.3: 1) DerSimonian-Laird random-effects models for pooled effect sizes (OR/RR/IRR); 2) Heterogeneity assessment via I^2^ statistics (subgroup analysis triggered at I^2^ ≥ 50%). Prespecified subgroups included methodological (medical record-confirmed vs. self-reported) and clinical dimensions (offspring age stratification). Sensitivity analyses comprised: 1) leave-one-out validation; 2) Egger's regression for publication bias (*P* < 0.1 significance); 3) competing risk models for mortality adjustment. All analyses reported 95% CI with *α* = 0.05 significance threshold.

## 3 Results

### 3.1 Literature screening process

**Figure 1:**
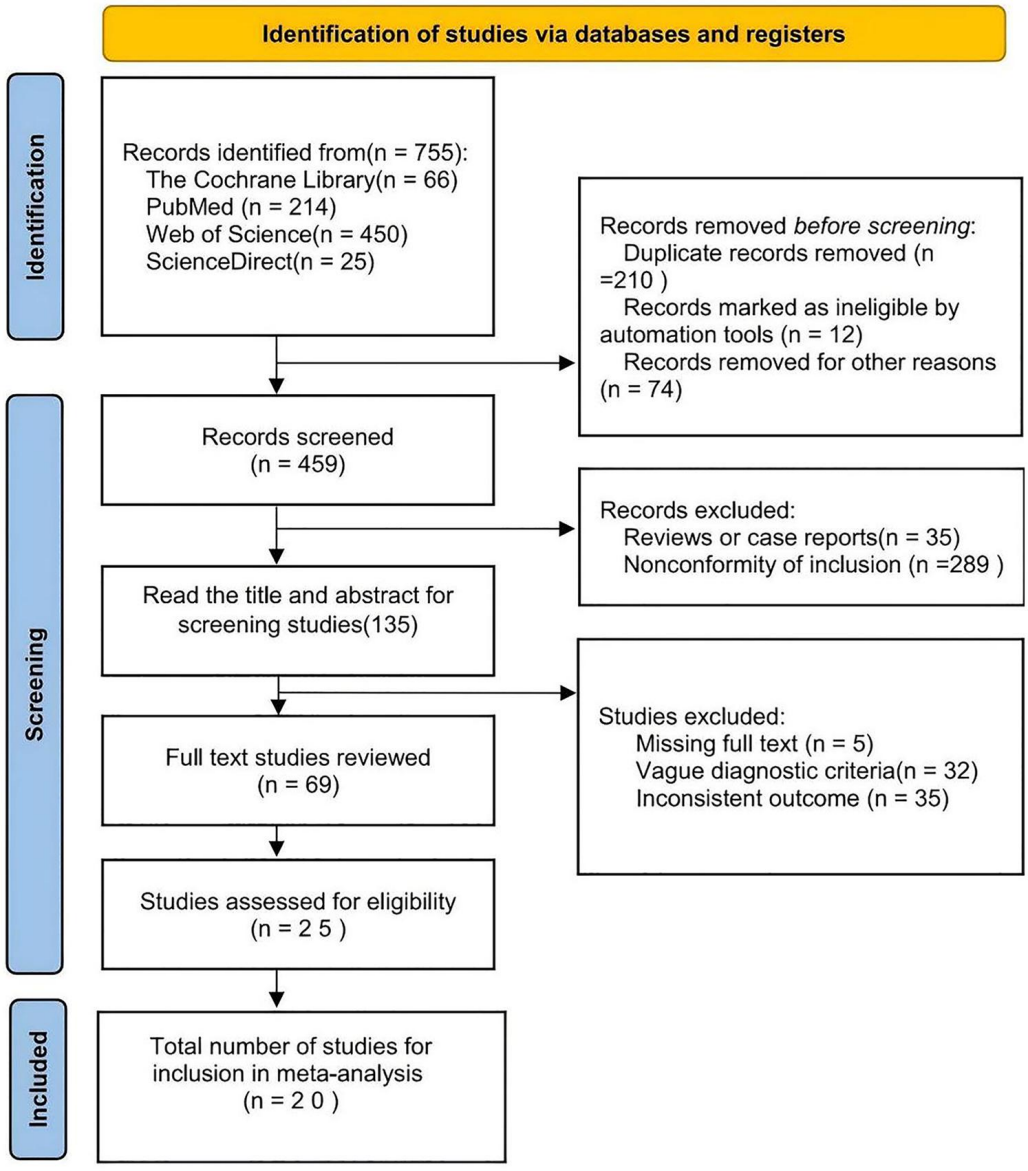
Flow diagram of the studies selected for inclusion in the systematic review and meta-analysis.

The literature screening process is detailed in Figure 1. Initially, studies were identified through databases and registries, yielding 755 articles in the initial search, including 66 from the Cochrane Library, 214 from PubMed, 450 from Web of Science, and 25 from ScienceDirect. Before screening, 210 duplicate articles and 12 articles marked as not meeting the criteria for other reasons were excluded. After de-duplication using EndNote X9, 459 articles remained. These 459 articles were then screened by title and abstract, excluding 35 review or case report articles and 289 articles that did not meet the inclusion criteria, leaving 135 articles for full-text assessment. During the full-text assessment, 5 articles with missing full texts, 32 articles with ambiguous diagnostic criteria, and 29 articles with inconsistent results[studies with internal contradictions in key outcomes (e.g., conflicting effect sizes for HDP and asthma in different subgroups) or unresolved discrepancies between exposure definitions and outcome measures (e.g., defining HDP without blood pressure thresholds) were excluded. Ultimately, 25 articles were eligible for study quality assessment. Using the JBI evidence-based tool, 5 articles with methodological flaws (4 with insufficient confounding adjustment and 1 with an unclear exposure definition) were excluded, and 20 studies were included for quantitative synthesis.

### 3.2 Characteristics of included studies

A total of 20 observational studies were included in this research (4–6, 8, 9, 11–22, 27, 28) (Table 1), with a combined sample size of 9,447,593 cases. The publication period ranged from 2002 to 2023, and the geographical distribution was mainly in Europe (15 studies, 75.0%), followed by Asia (4 studies, 20.0%) and North America (1 study, 5.0%). The study designs included 18 cohort studies (16 prospective cohort studies, accounting for 88.9% of the cohort studies; 2 retrospective cohort studies, accounting for 11.1%) and 2 cross-sectional studies (10.0%). The median sample size was 806,000 cases, and 5 studies (25.0%) had a sample size exceeding one million. Regarding exposure assessment, 75.0% of the studies (15 studies) confirmed the diagnosis of gestational hypertension disorders through medical records, while 25.0% (5 studies) relied on self-reports from pregnant women. For the diagnosis of childhood asthma, 50.0% (10 studies) were based on medical record verification, and 50.0% (10 studies) used standardized questionnaires. The follow-up age of the offspring ranged from the birth cohort (0–2 years) to young adulthood (26 years).

**Table 1:** Characteristics of the selected studies included in the systematic review and meta-analysis.

Author (year)	Country	Study design	Data source	Sample size (offspring)	Ascertainment of HDP/PE/PE	Ascertainment of asthma	Diagnosis age, year	Study quality
McKeever 2002 (PE)	UK	Cohort	GPRD	29,238	Medical records	Medical records	1	H
Nafstad 2003 (PE)	Norway	Cohort	RB	1,548,429	Medical records	Medical records	0–25	M
Wu 2009 (PE)	Denmark	Cohort	RB	1,618,481(identified) 1,545,443(eligible)	Medical records	Medical records	0–26	H
Aspberg 2010 (PE)	Sweden	Cohort	RB	1,338,319 (Cohort) 61,256 (Pediatric-antiasthmatics)	Medical records	Medical records	2–12	H
Algert 2011 (HDP)	Australia	Cohort	RB	240,511	Medical records	Medical records	2–5	H
Byberg 2014 (PE)	Norway	Cohort	MBRN	1,025	Medical records	F1:Medical records F2:Maternal report	10–12	M
Liu 2015 (PE)	Denmark	Cohort	RB	955,352 (identified) 923,533 (eligible)	Medical records	Medical records	3–18	H
Magnus 2016 (PE)	Norway	Cohort	RB, NMCCS	406,907 (Cohort) 45,028 (MoBa)	Medical records	Maternal report	7	H
Shaheen 2016 (GH/PE)	UK	Cohort	ALSPAC	13,978	Medical records	Maternal report	7.5	H
Stokholm 2017 (PE)	Denmark	Cohort	RB, COPSAC2000	411 (COPSAC2000) 1,698,638 (cohort)	Medical records	Maternal report	0–7/0–15	H
Pesce 2017 (HDP)	Italy	Retrospective	RCSS	3,907 (identified) 3,617 (eligible)	Maternal report	Maternal report	>6	M
Wilmink 2018 (GH/PE)	Netherlands	Cohort	GRS	6,168 (identified) 4,894 (eligible)	Medical records	Maternal report	10	H
Mirzakhani 2019 (PE)	USA	Cohort	VDAART	806	Medical records	Maternal report	0–3	H
Nahum 2020 (PE)	Israel	Cohort	PB	310,875 (identified) 253,808 (eligible)	Medical records	Maternal report	0–18	M
Henderson 2021 (HDP)	UK	Cohort	MCS	12,450	Maternal report	Maternal report	0–5	H
Byberg 2022 (PE)	Sweden	Cohort	RB	805,535 (identified) 779,711 (eligible)	Medical record	Medical records	0–2/>2	H
Kelly 2022 (HDP)	UK	Cohort	MCS	18,818 (identified) 13,089 (eligible)	Maternal report	Maternal report	3,5,7	M
Ma 2023 (GH)	China	Cohort	PHMPPHSS	193,471 (identified) 166,772 (eligible)	Medical records	Medical records	6–12	M
Papandreou 2023 (GH)	Greece	Retrospective	RCSS	7,038 (identified) 5,133 (eligible)	Medical records	Maternal report	2–5	M
Yang 2023 (GH/PE)	China	Cohort	RB	1,935,874 (identified) 1,919,023 (eligible)	Medical records	Medical records	>3	H

RB, registry-based; GPRD, general practice research database; MBRN, medical birth registry of Norway; NMCCS, the Norwegian mother and child cohort study; MoBa, Norwegian mother and child cohort study; ALSPAC, avon longitudinal study of parents and children; GRS, generation R study; COPSAC2000, copenhagen prospective studies on asthma in childhood2000; RCSS, retrospective cross-sectional; VDRRT, vitamin D antenatal asthma reduction trial; PB, population-based; MCS, millennium cohort study; PHMPPHSS, the physical health monitoring project of primary and high school students in Guangzhou; PE, preeclampsia; HDP, hypertensive disorders of pregnancy; GH, gestational hypertension; H, high quality; M, moderate quality.

The quality assessment results showed that among the 18 cohort studies, 72.2% (13 studies) were high-quality studies with a modified Newcastle-Ottawa Scale (mNOS) score of ≥7, and 27.8% (5 studies) were of moderate quality (with a score of 4–6). The main methodological limitation was insufficient adjustment for confounding factors. The two cross-sectional studies were both evaluated as moderate quality (meeting 6–8 criteria) by the Agency for Healthcare Research and Quality (AHRQ) checklist. These characteristics suggest that the included studies have a relatively high internal validity in the analysis of exposure-outcome associations, but attention should be paid to the potential information bias introduced by self-reported exposure measurement and questionnaire diagnosis.

### 3.3 The main results of the meta-analysis

**Figure 2:**
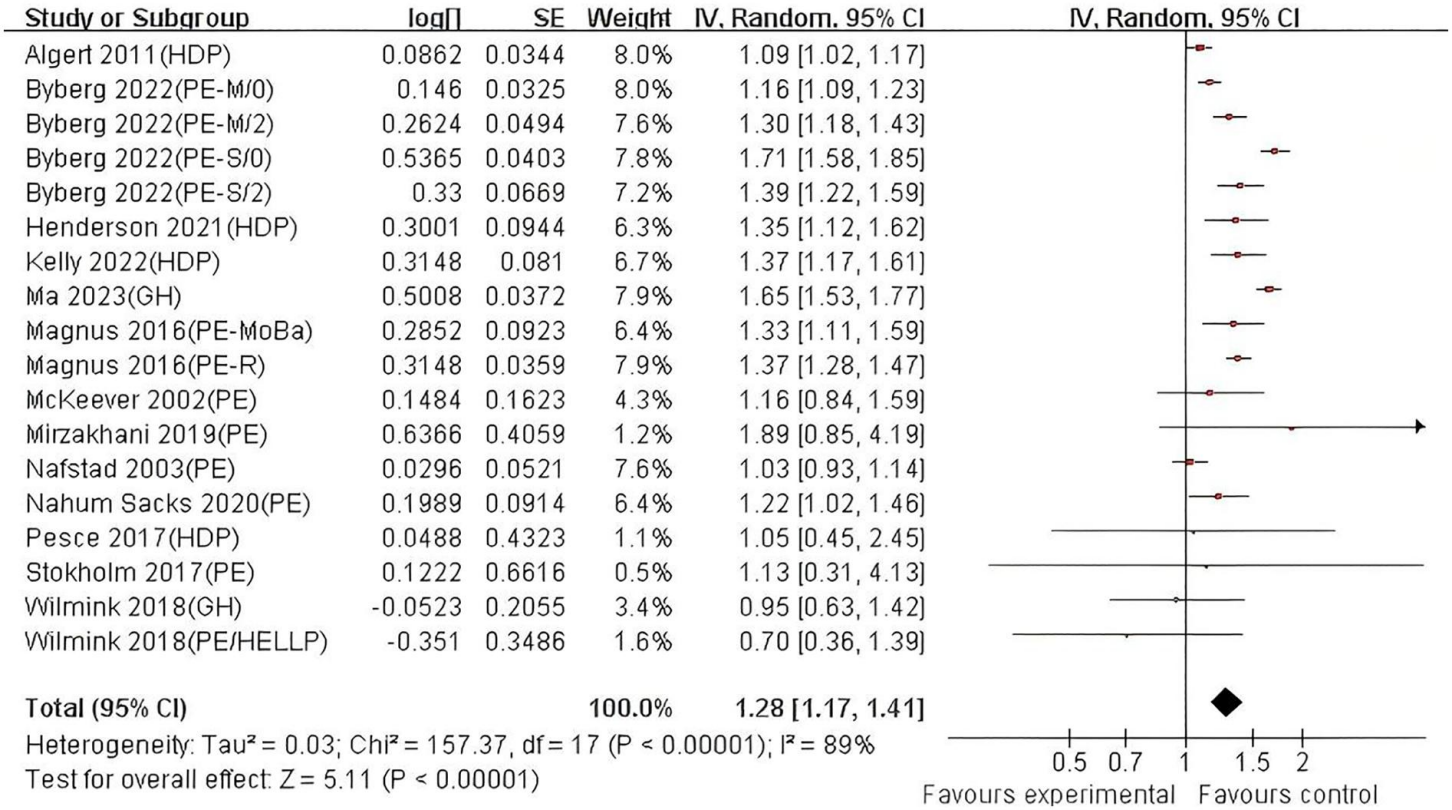
Forest plot of the association between hypertensive disorders in pregnancy and asthma in offspring (adjusted estimates*). HDP, hypertension disorders in pregnancy; PE, pre-eclampsia; PE-M, moderate pre-eclampsia;/0/2, Offspring under the age of 2 and over the age of 2; PE-S, severe pre-eclampsia; GH, gestational hypertension; HELLP, hemolysis, elevated liver enzymes and low platelets; PE-moBa, Norwegian Mother and Child Cohort Study of pre-eclampsia; PE-R, Registry-based study of pre-eclampsia. Note*: Adjusted for ≥3 confounders: maternal age, pre-pregnancy BMI, smoking, education, parity, gestational age, offspring sex, maternal asthma, diabetes, socioeconomic status.

**Figure 3:**
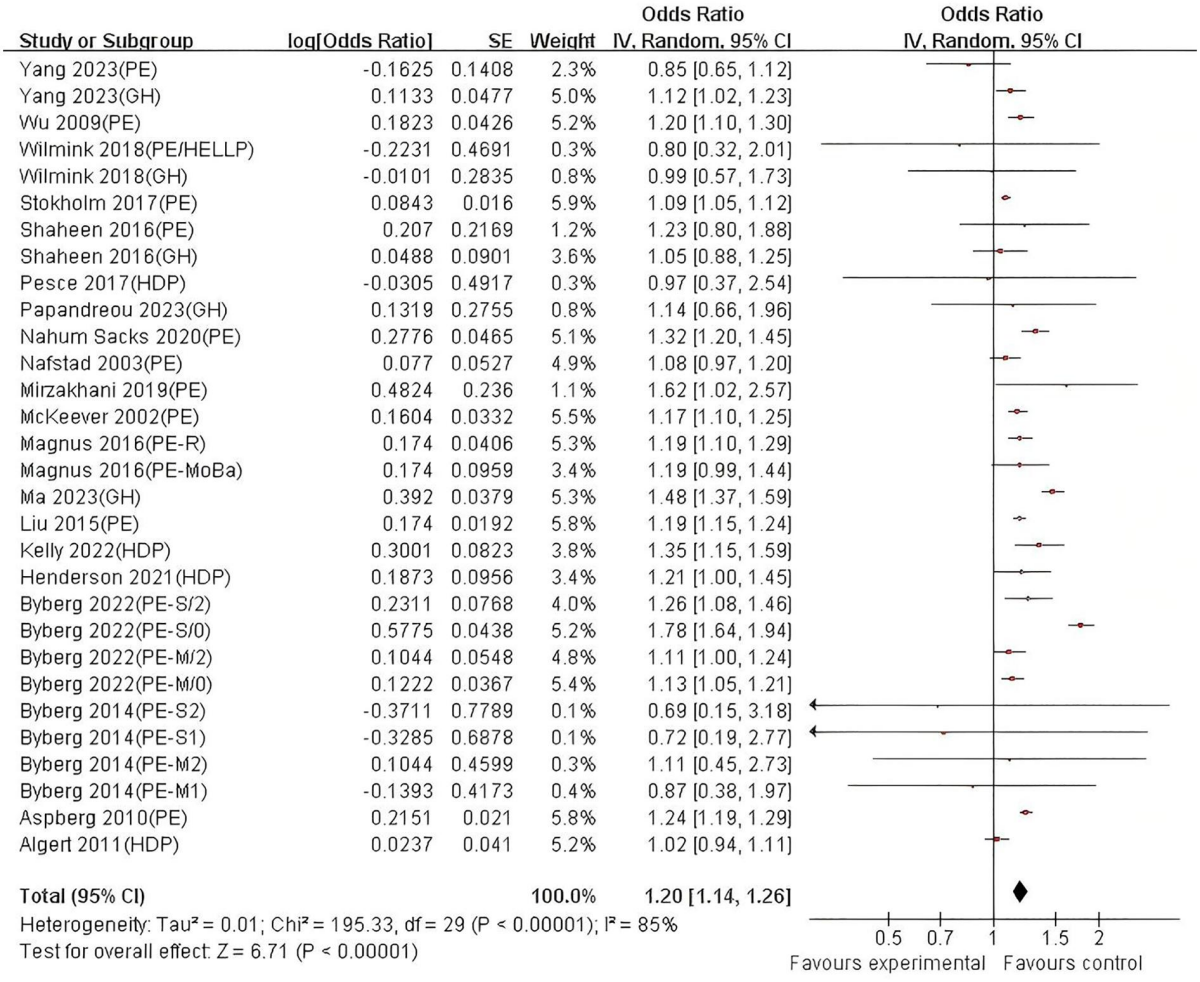
Forest plot of the association between hypertensive disorders in pregnancy and asthma in offspring (crude estimates). HDP, hypertension disorders in pregnancy; PE, pre-eclampsia; PE-M, moderate pre-eclampsia;/0/2, Offspring under the age of 2 and over the age of 2; PE-S, severe pre-eclampsia; GH, gestational hypertension; HELLP, hemolysis, elevated liver enzymes and low platelets; PE-moBa, Norwegian mother and child cohort study of pre-eclampsia; PE-R, registry-based study of pre-eclampsia.

The DerSimonian-Laird random-effects model was used to combine the effect sizes (Figures 2, 3). Twenty studies (*n* = 9,447,593) were included, showing that after full adjustment for confounding, exposure to hypertensive disorders in pregnancy significantly increased the risk of asthma in offspring (combined OR = 1.20, 95% CI: 1.14–1.26, *P* < 0.00001). The unadjusted model (crude effect) showed a similar association strength (OR = 1.28, 95% CI: 1.17–1.41), suggesting that residual confounding had a limited impact on the main effect. Heterogeneity analysis indicated high heterogeneity among studies (I^2^ > 80%, *P* < 0.0001), and further investigation of the sources of heterogeneity is needed. The assessment of publication bias showed good symmetry of the funnel plot (Egger's test: *t* = 1.02, *P* = 0.523, Supplementary E-Figure A), and the trim and fill method estimated that the number of missing studies was less than 3, suggesting a low risk of publication bias.

**Figure 4:**
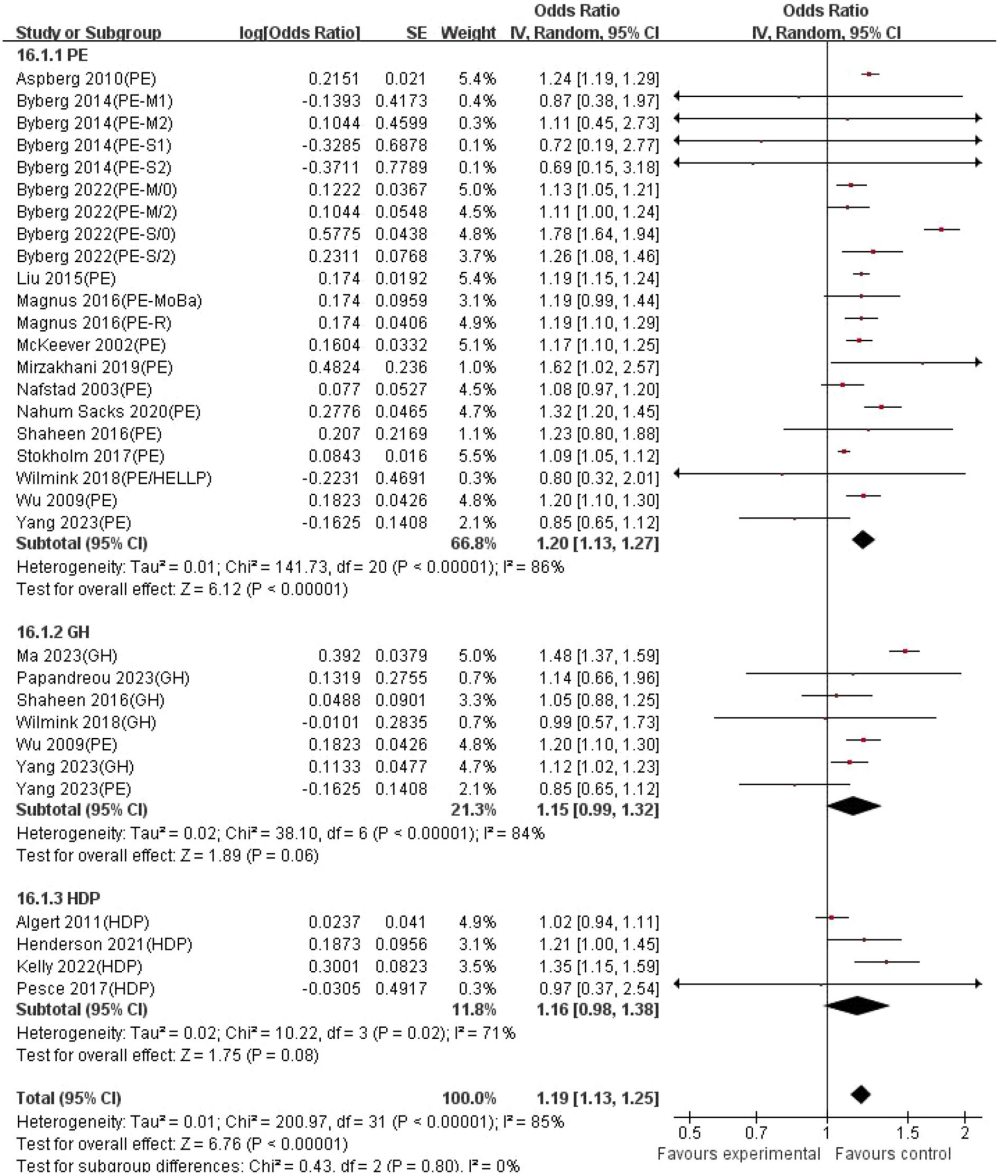
Forest plot of stratified analysis based on exposure type. (adjusted estimates*). HDP, hypertension disorders in pregnancy; PE, pre-eclampsia; PE-M, moderate pre-eclampsia;/0/2, Offspring under the age of 2 and over the age of 2; PE-S, severe pre-eclampsia; GH, gestational hypertension; HELLP, hemolysis, elevated liver enzymes and low platelets; PE-moBa, Norwegian mother and child cohort study of pre-eclampsia; PE-R, registry-based study of pre-eclampsia. Note*: Adjusted for ≥3 confounders: maternal age, pre-pregnancy BMI, smoking, education, parity, gestational age, offspring sex, maternal asthma, diabetes, socioeconomic status.

**Figure 5:**
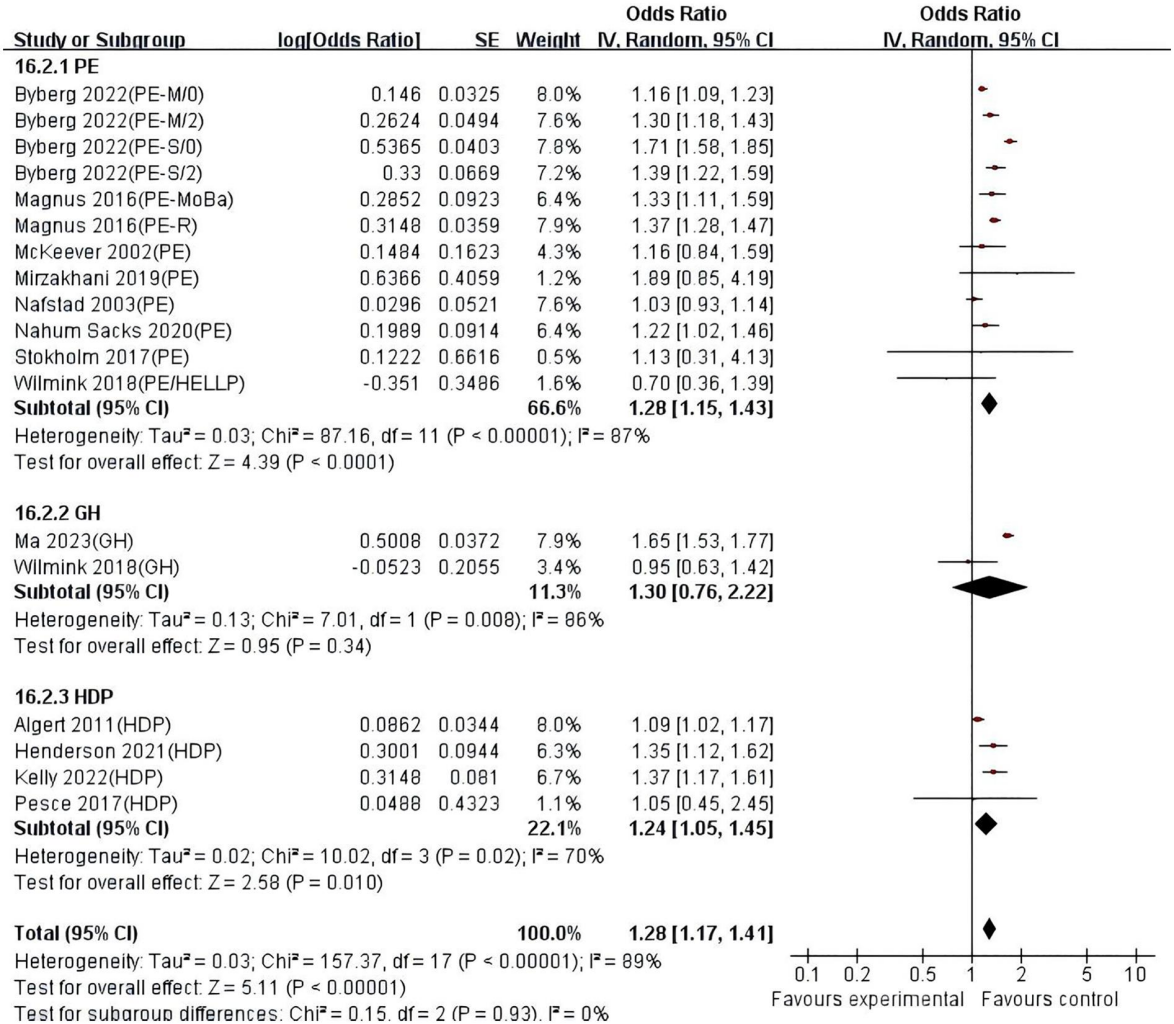
Forest plot of stratified analysis based on exposure type. (crude estimates). HDP, hypertension disorders in pregnancy; PE, pre-eclampsia; PE-M, moderate pre-eclampsia;/0/2, Offspring under the age of 2 and over the age of 2; PE-S, severe pre-eclampsia; GH, gestational hypertension; HELLP, hemolysis, elevated liver enzymes and low platelets; PE-moBa, Norwegian mother and child cohort study of pre-eclampsia; PE-R, registry-based study of pre-eclampsia.

Stratified analysis based on exposure type showed (Figures 4, 5) that in the model adjusted for confounding factors, the preeclampsia (PE) subgroup showed a significant association (combined OR = 1.20, 95% CI: 1.13–1.27; I^2^ = 86%), indicating high heterogeneity among studies, possibly due to differences in diagnostic criteria or population characteristics among different studies. The association in the gestational hypertension (GH) subgroup did not reach statistical significance (OR = 1.15, 95% CI: 0.99–1.32; I^2^ = 84%), and further validation with larger sample size studies is needed. The effect size of the combined exposure to hypertensive disorders in pregnancy (HDP) subgroup was 1.16 (95% CI: 0.98–1.38; I^2^ = 71%), with the confidence interval crossing the null value, indicating that heterogeneity may affect the stability of the results. In the unadjusted model, the association strength in the PE subgroup further increased (OR = 1.28, 95% CI: 1.15–1.43; I^2^ = 87%), and although the GH subgroup showed a wider confidence interval (OR = 1.30, 95% CI: 0.76–2.22; I^2^ = 86%), the effect size direction was consistent with the adjusted model, suggesting that confounding factors such as maternal underlying diseases may partially mediate the association. The HDP combined exposure subgroup remained significant in the unadjusted model (OR = 1.24, 95% CI: 1.05–1.45; I^2^ = 70%), indicating the independent effect of the exposure itself.

### 3.4 Subgroup and sensitivity analysis

#### 3.4.1 Subgroup analysis

Subgroup analysis data (Table 2) indicated that the association between exposure to hypertensive disorders in pregnancy (HDP) and the risk of asthma in offspring was consistent across different study designs: the pooled effect size from sibling-matched cohorts (OR = 1.24, 95% CI: 1.11–1.39) was similar to that from conventional cohort studies (OR = 1.18, 95% CI: 1.10–1.25), with similar effect magnitudes (I^2^ = 92% vs. 80% Stratified analysis by region showed significant associations in European populations (15 studies, OR = 1.20, 95% CI: 1.13–1.28) and North American populations (1 study, OR = 1.62, 95% CI: 1.02–2.57), while no statistical significance was reached in Asian populations (4 studies, OR = 1.16, 95% CI: 0.99–1.37) (*P* < 0.0001), suggesting potential regional heterogeneity (I^2^ = 93%). Stratification by age indicated a significantly increased risk in the infant and toddler period (0–2 years old group, OR = 1.27, 95% CI: 1.02–1.59), preschool age (7–12 years old group, OR = 1.22, 95% CI: 1.09–1.36), and mixed age groups (OR = 1.17, 95% CI: 1.11–1.24), but no statistical association was observed in the 2–6 years old group (OR = 1.03, 95% CI: 0.95–1.11). Notably, quality control of the studies showed that high-quality studies (12 studies, OR = 1.18) and medium-quality studies (8 studies, OR = 1.25) had consistent effect directions. After adjusting for study design heterogeneity factors, both singleton studies (OR = 1.25, 95% CI: 1.17–1.34) and non-singleton studies (OR = 1.09, 95% CI: 1.06–1.12) remained statistically significant, suggesting that the impact of HDP on offspring asthma is independent of confounding factors such as multiple pregnancies.

**Table 2:** Subgroup analysis: HDP and offspring asthma.

Variable	*N*	OR	95% CI	I^2^ (%)	*P* (Heterogeneity)
Sibling design					
Yes	4	1.24	(1.11, 1.39)	92	<0.0001
No	16	1.18	(1.1, 1.25)	80	<0.0001
Quality					
High	12	1.18	(1.11, 1.26)	87	<0.0001
Moderate	8	1.25	(1.11, 1.42)	64	0.0003
Location					
Europe	15	1.2	(1.13, 1.28)	83	<0.0001
Asia	4	1.16	(0.99, 1.37)	93	<0.0001
America	1	1.62	(1.02, 2.57)	–	–
Offspring age					
0–2	2	1.27	(1.02, 1.59)	95	<0.0001
2–6	2	1.03	(0.95, 1.11)	0	0.7
7–12	6	1.22	(1.09, 1.36)	60	0.004
Other	10	1.17	(1.11, 1.24)	79	<0.0001
Only singlton					
Yes	13	1.25	(1.17, 1.34)	87	<0.0001
No	7	1.09	(1.06, 1.12)	0	0.92
Exposure assessment way					
Medical records	15	1.19	(1.12, 1.26)	87	<0.0001
Maternal report	5	1.34	(1.19, 1.51)	0	0.84
Outcome assessment way					
Medical records	10	1.2	(1.11, 1.29)	90	<0.0001
Maternal report	10	1.2	(1.11, 1.29)	53	0.008
Maternal asthma adjusted					
Yes	13	1.18	(1.1, 1.26)	85	<0.0001
No	7	1.29	(1.18, 1.4)	68	0.003
Maternal smoking adjusted					
Yes	11	1.25	(1.16, 1.34)	84	<0.0001
No	9	1.13	(1.06, 1.21)	69	0.0004

#### 3.4.2 Sensitivity analysis

The robustness of the results was verified by successively excluding individual studies. The combined OR fluctuated within the range of 1.18–1.22 (directional consistency test *P* < 0.001), indicating that the results were not sensitive to extreme values. After adjusting for competing events such as offspring death using the competing risk model, the subdistribution hazard ratio (OR = 1.19, 95% CI: 1.10–1.29) was consistent with the main effect. Further converting the risk ratio (RR) and incidence rate ratio (IRR) to odds ratios (OR), the combined effect size changed minimally (ΔOR < 0.02), confirming that the methodological conversion had no substantial impact on the conclusion. Additionally, the symmetry of the funnel plot (Egger's test *P* = 0.523) and the trim-and-fill method estimation (number of missing studies <3) both indicated a low risk of publication bias. The above analyses collectively suggest that the association between HDP and offspring asthma is statistically robust and clinically consistent, but potential sources of heterogeneity in Asian populations and children aged 2–6 years should be noted. The above results provide high-level evidence supporting the suggestive of a potential causal link between HDP exposure and offspring asthma, but further mechanism studies are needed to clarify the biological pathways and clinical phenotype specificity underlying the heterogeneity.

## 4 Discussion

This study systematically revealed the dose-response relationship between hypertensive disorders in pregnancy (HDP) and the risk of asthma in offspring by integrating 20 global observational studies (*n* = 9,447,593). The effect intensity was robust across different clinical subtypes, geographical regions, and conditions of confounding control. The following discussion is presented from aspects such as molecular mechanisms, research heterogeneity, advantages and limitations, as well as clinical and research implications.

### 4.1 Multidimensional analysis of molecular pathological mechanisms

HDP may affect the development of the respiratory system in offspring through a triple cascade mechanism: First, abnormal remodeling of the placenta-fetus interface signals, characterized by a significantly elevated ratio of soluble fms-like tyrosine kinase-1 (sFlt-1) to placental growth factor (PlGF) in preeclampsia, inhibits the vascular endothelial growth factor (VEGF) pathway, thereby impeding the maturation of alveolar type II epithelial cells. Animal models have shown that this mechanism can significantly reduce the expression of surfactant protein-C (SP-C) (29–31). Second, epigenetic reprogramming, based on epigenome-wide association studies (EWAS), shows that the promoter region of the FOXP3 gene, which regulates the differentiation of regulatory T cells (Treg), is hypermethylated in the umbilical cord blood DNA of HDP-exposed offspring, which may promote asthma by enhancing Th2 immune polarization (32, 33). Third, compensatory accelerated lung maturation (pulmonary acceleration index ≥2.5) caused by fetal growth restriction (FGR) may lead to thickening of the airway basement membrane, thereby increasing the risk of airway hyperresponsiveness (34, 35). Recent human cohort studies further support the association between placental sFlt-1 levels and asthma in offspring, providing direct clinical evidence for the mechanism (36–38).

### 4.2 Heterogeneity analysis

Although overall analysis indicated a significant association between HDP exposure and offspring asthma risk, subgroup analyses revealed notable heterogeneity: no significant association was observed in the 2–6 years age group or Asian subgroups. This divergence may stem from multidimensional mechanisms:

1. Developmental Stage-Specific and Environmental Interactions

In children aged 2–6 years, wheezing is often transient and triggered by viral infections, which may overlap with asthma symptoms, leading to misclassification bias. This misclassification could weaken the observed association between HDP and asthma in this age group, as wheezing cases without true asthma may dilute the effect size (39, 40). Environmental exposures during the 2–6 year age period (such as PM2.5 and allergens) may override prenatal effects through epigenetic reprogramming (e.g., FOXP3 methylation) (41–43). In Asian subgroups (44, 45), vitamin D sufficiency might mitigate HDP risk through placental oxidative stress modulation (46), but existing evidence has not systematically evaluated the regulatory effects of nutritional or genetic factors.

1. Methodological and Regional Variability

Asthma diagnosis in 2–6-year-olds relies on clinical symptoms (GINA guidelines discourage routine spirometry) (47–49), potentially introducing misclassification. Asian heterogeneity may arise from cultural factors (breastfeeding rates >80% vs. 65% in Europe) (50–52) or PM2.5 compositional differences (53–56), unadjusted in subgroup analyses. Moreover, 73.7% of included studies were European, with only 21.1% Asian representation and no low-middle-income country data, limiting generalizability. Ni et al. (57) reported that their global analysis of 200 countries confirms MHD's association with early childhood asthma/atopic dermatitis in 1–4-year-olds, aligning with our meta-analysis. Notably, their observation of age-dependent attenuation (weaker in older children) supports our null finding in 2–6-year-olds, while their extension to atopic dermatitis and GAM adjustment for socioeconomic factors strengthen the external validity of our conclusions.

1. Mechanistic and Temporal Gaps

The follow-up time may be insufficient to detect delayed effects. Current mechanistic evidence predominantly from animal models requires human validation of placental biomarkers (miR-155) and peripheral epigenetic markers (e.g., FOXP3 methylation).

Future multi-center cohorts should integrate multimodal assessments (e.g., lung function + biomarkers) and Mendelian randomization to dissect gene-environment interactions. Establishing transnational biobanks will advance our understanding of developmental and region-specific mechanisms, informing targeted prevention strategies.

### 4.3 Strengths and limitations

The strengths of this study include: (1) Incorporating 20 large-sample studies (total sample size >9.4 million), the system evaluates the dose-response relationship between HDP subtypes and asthma risk; (2) Utilizing strict quality assessment tools (such as mNOS, AHRQ) to ensure the methodological rigor of the included studies; (3) Confirming the robustness of the results through sensitivity analysis and trim-and-fill method. However, limitations still need to be noted: (1) Most studies were observational designs, making it impossible to rule out residual confounding (such as paternal asthma history, maternal nutritional status during pregnancy); (2) There is a scarcity of data from Asian populations (4 studies), which may underestimate the effect of HDP in non-European populations; (3) Self-reported asthma diagnosis may miss mild cases, leading to bias in effect estimates; (4) Residual confounding (e.g., paternal asthma history, maternal vitamin D levels) cannot be fully excluded. Mendelian randomization or interventional studies are needed to confirm causality; (5) Subtype-specific analyses (e.g., allergic vs. exercise-induced asthma) were not feasible due to inconsistent reporting across studies. Future research should explore HDP differential impact on asthma phenotypes.

### 4.4 Clinical and research implications

The findings of this study provide evidence-based guidance for the health management of offspring of HDP patients. It is recommended that clinical practice should conduct long-term respiratory function monitoring for the offspring of HDP pregnant women, especially during infancy and school age. Future research should focus on: (1) HDP subtype-specific mechanisms, such as the differences in placental transcriptomes between GH and PE; (2) Gene-environment interactions, such as whether maternal vitamin D levels modify the association between HDP and asthma; (3) Intervention strategies, such as whether low-dose aspirin treatment during pregnancy can reduce the risk of asthma in offspring.

## 5 Conclusion

This study confirmed that exposure to hypertensive disorders in pregnancy (especially pre-eclampsia) is significantly associated with an increased risk of asthma in offspring, and this association is age- and region-specific. Although the underlying mechanisms require further exploration, the results suggest that HDP may be a critical window for programming respiratory tract development in offspring. By integrating multi-omics data and prospective intervention studies, it is expected that the biological pathways leading to asthma due to HDP can be clarified in the future, and targets for early prevention can be provided.

## Data Availability

The original contributions presented in the study are included in the article/Supplementary Material, further inquiries can be directed to the corresponding author.
