# Supplementary material for: Maternal hypertensive disorders during pregnancy and their link to childhood asthma: a systematic review and meta-analysis
Source: Front Pediatr. 2025 Dec 8;13:1659105. doi: 10.3389/fped.2025.1659105 (PMC12722935; doi:10.3389/fped.2025.1659105)
Supplement: Supplementary file 2 [file Datasheet2.docx]

**Maternal Hypertensive Disorders During Pregnancy and Their**

**Link to Childhood Asthma: A Systematic Review and**

**Meta-Analysis**

**search strategy**

**MeSH**:Hypertension, Pregnancy-Induced **free-text keywords**：

Hypertension, Pregnancy Induced Pregnancy-Induced Hypertension Gestational Hypertension

Hypertension, Gestational

Pregnancy Induced Hypertension Hypertensions, Pregnancy Induced Induced Hypertension, Pregnancy Induced Hypertensions, Pregnancy Transient Hypertension, Pregnancy Hypertension, Pregnancy Transient Pregnancy Transient Hypertension **MeSH**:Asthma

**free-text keywords**：

Asthmas

Asthma, Bronchial Bronchial Asthma

**Search strategy**： (((((((((((((Hypertension, Pregnancy-Induced[Title/Abstract]) OR (Hypertension, Pregnancy Induced[Title/Abstract])) OR (Pregnancy-Induced Hypertension[Title/Abstract])) OR (Gestational Hypertension[Title/Abstract])) OR (Hypertension, Gestational[Title/Abstract])) OR (Pregnancy Induced Hypertension[Title/Abstract])) OR (Hypertensions, Pregnancy Induced[Title/Abstract])) OR (Induced Hypertension, Pregnancy[Title/Abstract])) OR (Induced Hypertensions, Pregnancy[Title/Abstract])) OR (Transient Hypertension, Pregnancy[Title/Abstract])) OR (Hypertension, Pregnancy Transient[Title/Abstract])) OR (Pregnancy Transient Hypertension[Title/Abstract])) OR (Hypertension, Pregnancy-Induced[MeSH Terms])) AND (((((Asthma[MeSH Terms]) OR (Asthma[Title/Abstract])) OR (Asthmas[Title/Abstract])) OR (Asthma, Bronchial[Title/Abstract])) OR (Bronchial Asthma[Title/Abstract]))

**Result：**

PUBMED：214

Cochrane Library：66

Web of Science：450

Elsevier ScienceDirect：25
