# Supplementary material for: Maternal hypertensive disorders during pregnancy and their link to childhood asthma: a systematic review and meta-analysis
Source: Front Pediatr. 2025 Dec 8;13:1659105. doi: 10.3389/fped.2025.1659105 (PMC12722935; doi:10.3389/fped.2025.1659105)
Supplement: Supplementary file 3 [file Table1.docx]

**E-Figure A**
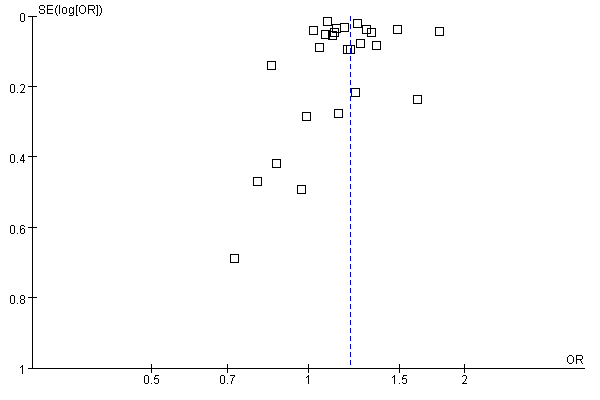
**.** Funnel plot of the combined assessment of bias risk of studies included in the meta-analysis
